# Is It a Gut Feeling? Bodily Sensations Associated With the Experience of Valence and Arousal in Patients With Inflammatory Bowel Disease

**DOI:** 10.3389/fpsyt.2022.833423

**Published:** 2022-04-22

**Authors:** Konstantina Atanasova, Tobias Lotter, Robin Bekrater-Bodmann, Nikolaus Kleindienst, Wolfgang Reindl, Stefanie Lis

**Affiliations:** ^1^Department of Clinical Psychology, Central Institute of Mental Health, Medical Faculty Mannheim, Heidelberg University, Mannheim, Germany; ^2^Department of Medicine II, Medical Faculty Mannheim, Heidelberg University, Mannheim, Germany; ^3^Department of Psychosomatic Medicine, Central Institute of Mental Health, Medical Faculty Mannheim, Heidelberg University, Mannheim, Germany

**Keywords:** inflammatory bowel disease, emotional awareness, bodily sensations, arousal, valence, emotion perception

## Abstract

**Background:**

Previous studies have shown dysfunctional emotion processing in patients with inflammatory bowel diseases (IBD), characterized by a hypersensitivity to negative emotions and a hyposensitivity to positive emotions. Models of emotion processing emphasize the importance of bodily sensations to the experience of emotions. Since there have been no studies on whether emotion-associated bodily sensations are changed in IBD, we investigated the experience of bodily sensations related to valence and arousal, together with their links to emotional awareness, as one domain of interoceptive sensibility relevant to emotion processing.

**Methods:**

Using a topographical self-report measure, 41 IBD patients in clinical remission and 44 healthy control (HC) participants were asked to indicate where and how intensely in their body they perceive changes when experiencing emotions of positive and negative valence, as well as relaxation and tension. Additionally, we used self-report questionnaires to assess emotional awareness as one domain of an individual’s interoceptive sensibility, gastrointestinal-specific anxiety (GSA), and psychological distress.

**Results:**

Patients with IBD reported higher emotional awareness but lower intensities of perceived changes in their bodily sensations related to valence and arousal of emotional processing. IBD patients reported less intense bodily activation during positive emotions and less intense bodily deactivation during negative emotional states in comparison to HC participants. Higher emotional awareness and psychological distress were linked to stronger experiences of emotion-related bodily sensations in IBD patients.

**Conclusion:**

Inflammatory bowel diseases patients exhibited alterations in how they link bodily sensations to their emotional experience. Such persistent changes can affect a patient’s wellbeing and are related to higher levels of anxiety and depression among IBD patients, even in remission.

## Introduction

Patients with inflammatory bowel disease (IBD) report poorer quality of life, including impairments in their physical and mental wellbeing, than healthy individuals (1). IBD is often accompanied by increased attention to and anxiety about gastrointestinal sensations, resulting in greater psychological distress (2). Psychological distress and biased emotion experience have been shown to be subjectively and objectively related to disease activity (3, 4). These relationships are consistent with the biopsychosocial model of illness, according to which the disease course affects and is affected by biological, social, and psychological factors, such as emotions and cognitions (5). The perception of bodily sensations significantly influences the experience of stress and emotions (6, 7). Thus, chronic conditions associated with altered visceral and interoceptive processing, such as gastrointestinal inflammation, might result in impaired emotional functioning, potentially affecting patients’ quality of life and psychological wellbeing (8, 9).

Studies on emotional experience in IBD have pointed toward the presence of emotional dysfunctions, characterized by decreased sensitivity to positive emotional cues (9, 10), increased sensitivity to negative emotions and increased arousal during emotional experience (11). Moreover, neuroimaging findings have indicated a reduced activation within the limbic system in response to positive emotional stimuli in this patient group (10). In IBD, biological and emotional factors interact trough the brain-gut axis, as the limbic system communicates with the intrinsic neural network of the gastrointestinal tract *via* the sympathetic and parasympathetic nerves (12). Alterations in these brain regions have been associated with impaired regulation of the sympathetic and parasympathetic stress response in IBD patients, promoting the proinflammatory activity (4, 13). Thus, poor emotional functioning is thought to negatively affect the course of the disease and to be linked to greater psychological distress in IBD.

According to dimensional models of emotion, emotions are arranged in a two-dimensional space defined by the dimensions of valence and arousal (14). Valence refers to the hedonic value of an emotion, that is, the subjective feeling of pleasantness or unpleasantness. Arousal refers to the subjective state of feeling relaxation or tension (15) and represents the level of autonomic activation that an emotions elicits. Both dimensions influence peoples’ approach–avoidance behavior: high positive arousal states trigger the appetitive motivational system, while low negative arousal states are experienced as not rewarding and are therefore avoided (16). An individual’s level of emotional arousal is modulated by interoceptive processes (17). Part of an individual’s interoceptive abilities is the ability to link the conscious experience of emotions to the physiological sensations perceived in different regions of the body (18). A study by Nummenmaa et al. (19) showed that the experience of emotions is linked to topographical patterns of activation and deactivation perceived in the body, suggesting their representation in the somatosensory system (19, 20). Emotional awareness constitutes a distinct feature of one’s interoceptive sensibility and describes the ability to attribute specific bodily sensations to physiological manifestations of emotions (21). Although it has been shown that IBD patients exhibit certain alterations in both emotion and interoceptive processing (22), no study has investigated the role of interoceptive sensibility in the embodiment of valence and arousal among patients with IBD.

The present study aims to contribute to the understanding of how emotional valence and arousal are subjectively experienced in the body among patients with IBD and whether emotion-related sensations, possibly altered by increased anxiety about gastrointestinal sensations (23), are linked to patients’ emotional awareness. Using a topographical self-report measure adapted from Nummenmaa et al. (19), we assessed perceived changes in bodily sensations associated with experiences of positive and negative valence, as well as tension and relaxation, among patients with IBD and healthy control participants. We hypothesized that (I.) IBD patients would report stronger bodily sensations for negative emotions and more attenuated sensations for positive emotions than healthy control participants. We also expected that (II.) interoceptive sensibility and gastrointestinal-specific anxiety (GSA) would be related to the severity of alterations in emotion-related bodily sensations in IBD, particularly for negative emotions. To investigate whether these changes show distinct topographical patterns, especially in body areas strongly related to IBD symptoms, we explored whether IBD patients exhibit altered bodily sensations in the abdomen related to the experience of valence and arousal.

## Materials and Methods

### Participants

A total of 86 individuals between 18 and 65 years of age participated in the study. Of these, 42 met the diagnostic criteria for IBD, and 44 were healthy control (HC) participants. We excluded one participant from the IBD group because of a lack of understanding of the instructions. Thus, the final sample consisted of 41 IBD patients (20 female, 21 male) and 44 HC participants (30 female, 14 male; Table 1). Outpatients attending the IBD outpatient unit at the University Medical Center Mannheim were recruited during the routinely control visits and invited to participate in the study. Patients, who met the inclusion and exclusion criteria and provided written informed consent, took part in the study after the physical examination. Overall, 29 patients with Crohn’s disease (CD) and 12 patients with ulcerative colitis (UC) in clinical remission were included. We focused on examining IBD patients in clinical remission to avoid potentially confounding effects of active disease symptoms on the measures of emotional experience. Furthermore, we were interested in examining whether alterations in emotion experience could also be observed during clinical remission, potentially affecting a patient’s wellbeing even when acute disease symptoms have subsided. CD patients exhibited a mean Harvey–Bradshaw Index score (24) of 1.76 ± 1.83 (range: 0–16), and UC patients exhibited a mean Partial Mayo Score (25) of 1.58 ± 1.51 (range: 0–7). The average age for disease onset reported by the patients was 25.54 ± 10.24 years. The mean disease duration reported by the patients was 14.65 ± 12.64 years. Diagnostic procedures and gastroenterological examinations were carried out on all patients by fully trained physicians specialized in the care of patients with IBD. Exclusion criteria were biological signs of disease activity (fecal calprotectin level > 200 μg/g, C-reactive protein > 50 mg/L, HBI score > 5, Partial Mayo score > 3), current use of corticosteroids, use of psychotropic medications, and current or past neurological or psychiatric diseases. For further details on sample characteristics, see Table 1. Details on treatment medication, comorbidities, previous surgeries, and extraintestinal manifestations were collected from medical reports and are provided in Supplementary Table 1.

**TABLE 1:** Sample characteristics.

	IBD (M ± SD)	HC (M ± SD)	Test-statistics	*p*-value
**Demographics**				
Age	40.20 ± 13.87	36.11 ± 12.29	1.44^a^	0.154
Sex (female/male)	20/21	30/14	3.30^b^	0.069
Years of education	12.32 ± 3.01	12.84 ± 2.40	−0.89^a^	0.376
**Affective state**				
STAI anxiety (state)	33.13 ± 5.28	32.66 ± 8.20	0.31^a^	0.754
SAM-valence	3.75 ± 0.98	3.77 ± 0.85	−0.11^a^	0.911
SAM-arousal	2.34 ± 1.00	1.98 ± 0.86	1.78^a^	0.079
SAM-dominance	3.63 ± 0.98	3.68 ± 0.66	−3.41^a^	0.734
**Psychological distress**				
GSI	10.42 ± 8.74	4.42 ± 4.91	3.66^a^	0.001**
Somatization	3.97 ± 3.51	0.74 ± 1.27	5.31^a^	<0.001***
Depression	3.00 ± 3.80	1.58 ± 2.81	1.85^a^	0.068(*)
Anxiety	3.78 ± 3.65	2.09 ± 2.22	2.45^a^	0.017*
**Interoceptive sensibility**				
Emotional awareness	3.32 ± 0.92	2.77 ± 0.88	0.56^a^	0.006**
**Gastrointestinal-specific anxiety**				
VSI	27.42 ± 15.28	–	–	–

*IBD, inflammatory bowel diseases group; HC, healthy control group; STAI, State–Trait Anxiety Inventory; SAM, Self-Assessment Manikin; GSI, General symptom index; Emotional Awareness: subscale of the Multidimensional Assessment of Interoceptive Awareness; VSI, Visceral sensitivity index.*

(*)p < 0.10, *p < 0.05, **p < 0.01, ***p < 0.001.

*^a^t-value.*

*^b^Chi^2^.*

Exclusion criteria for the HC group were chronic medical conditions, chronic medication intake, use of psychotropic medication, and current or past neurological or psychiatric diseases, as well as general gastrointestinal complaints during the 4 weeks prior the experiment. The study was approved by the Ethics Committee of the Medical Faculty Mannheim at Heidelberg University, and all participants gave their written informed consent before participating in the study. Please note that a subsample of participants from the IBD and the healthy control group in this study was also included in another manuscript on interoception and emotion perception published by our group [see Atanasova et al. (22)].

All of the participants completed a battery of psychometric questionnaires assessing psychological distress, interoceptive sensibility, and gastrointestinal-specific anxiety. The participants then performed a computer-based task, and changes in the participants’ bodily sensations when experiencing valence and arousal were assessed.

## Questionnaires

### Brief Symptom Inventory

To characterize the participants, we assessed psychological distress using the Brief Symptom Inventory (BSI-18) (26). BSI-18 is a self-report measure of somatization, depression, and anxiety. Scores on three subscales with six items each are combined into a global symptom severity index (GSI). GSI scores range from 0 to 72 points, and scores on each of the three subscales range from 0 to 24 points, with higher scores indicating higher symptom severity. Cronbach’s alphas for the 18 items were 0.86 for the IBD group and 0.81 for the HC group.

### Affective State

Prior to testing, the current affective states of the participants were evaluated using the state version of the State–Trait Anxiety Inventory [STAI; Spielberger et al. (27)]. The STAI is a 20-item scale that measures subjective feelings of apprehension, tension, nervousness, worry, and activation of the autonomic nervous system on a four-point Likert scale. Item scores are added to obtain the scale total score (which ranges from 20 to 80), with higher scores indicating higher anxiety. Internal consistency was good, with Cronbach’s α = 0.76 for the IBD group and 0.90 for the HC group. Arousal, valence, and dominance levels were assessed using the Self-Assessment Manikin [SAM; Bradley and Lang (28)]. The SAM is a non-verbal pictorial assessment technique that measures valence/pleasure, perceived arousal, and perceptions of dominance on a nine-point Likert scale. Higher scores indicate more positive valence, higher arousal, and higher perceived dominance.

### Visceral Sensitivity Index

Gastrointestinal-specific anxiety was measured using the Visceral sensitivity index [VSI; Labus et al. (29)]. The 15-item scale assesses worry, fear, vigilance, sensitivity, and avoidance, as well as gastrointestinal-related cognitions and behaviors. Items are scored on a reversed six-point scale. The overall VSI score ranges from 0 to 75, with higher scores indicating more severe GSA. VSI was developed specifically for patients with functional gastrointestinal disorders and is not suitable for assessment of healthy individuals. For that reason, we utilized this self-report measure for the IBD group only. The internal consistency of this scale for the current IBD sample was good, with Cronbach’s α = 0.89.

### Multidimensional Assessment of Interoceptive Awareness

We assessed *Emotional Awareness* as the feature of interoceptive sensibility that is most strongly related to the experience of emotions in the body, as it comprises an individual’s awareness that certain physical sensations are the sensory aspects of emotional states. For this purpose, we used the Multidimensional Assessment of Interoceptive Awareness [MAIA; Mehling et al. (21)] and its *Emotional Awareness* subscale, which comprises five items assessed on a six-point Likert scale. The subscale score is calculated as the mean item score, with higher scores indicating higher emotional awareness. *Emotional Awareness* exhibited acceptable internal consistency, with Cronbach’s α = 0.76 for the IBD group and 0.86 for the HC group. For further details on the MAIA and its subscales, see Supplementary Material.

## Experimental Procedure

Participants were asked to indicate changes in their bodily sensations when they experienced emotional states of different valence (positive/negative) and arousal (relaxation/tension) during everyday life, using a topographical self-report method adapted from Nummenmaa et al. (19). Initially, participants were familiarized with the task and instructed to evaluate which bodily regions they typically felt as being activated or deactivated when experiencing a particular emotional state. Thus, the task did not involve inducing actual emotions by experimental manipulation. To indicate these changes, participants were asked to color the body areas where they perceived sensations of activation and deactivation by selecting a color from a nine-point color bar ranging from blue (−4 = “very strong deactivation”) to red (+4 = “very strong activation”) (see Supplementary Figure 1). Participants indicated their responses by painting the bodily regions using successive strokes on the presented body templates. If they did not perceive any changes in some parts of the body, participants were instructed to leave these uncolored. The four instructions (positive emotions, negative emotions, relaxation, and tension) were presented in a pseudorandomized order. The task was presented on a 14″ computer screen. The front and back body templates comprised 366 × 195 pixels, and the diameter of the painting tool was 13 pixels. The task was programmed using the Presentation^®^ software (Version 20.1, Neurobehavioral Systems, Inc., Berkeley, CA, United States).^1^

## Dependent Variables

For each participant and trial, color code values (ranging from -4 to + 4) were stored in a numeric array for each pixel of the body templates and spatially smoothed by averaging the color code value of each pixel and its surrounding pixels (±3 pixels).

To assess changes in bodily sensations, we calculated (a) a change score representing the average of the absolute values of the pixels and (b) a change score across those pixels that indicated either a deactivation or an activation. This procedure was conducted for all pixels of the body templates to derive a global whole-body score, as well as for different regions of interest (ROIs). The ROIs were predefined based on the findings of Nummenmaa et al. (19) and included the head, chest, arms, abdomen, legs, and back as separate body regions (see Supplementary Figure 2). Whole-body and ROI analyses were performed using custom pipelines run with MATLAB 2020a (The MathWorks, Inc., Natick, MA, United States).

## Data Analysis

The dependent variables were analyzed separately for the experience of valence and arousal using mixed-effects analysis-of-variance designs (mixed-effects ANOVAs). Testing for normality of residuals (Kolmogorov–Smirnov test, visual inspection of Q–Q plots) revealed that the normality assumptions underlying parametric analyses were violated. Therefore, a non-parametric approach was employed, using a rank-aligned ANOVA (30, 31). Global change scores were analyzed in 2 × 2 designs with the between-subjects factor “group” (IBD/HC) and an additional experimental factor, i.e., “valence” (positive/negative) in one case and “arousal” (relaxation/tension) in another. To analyze more specifically the changes in activation and deactivation, we extended these designs by an additional within-subject “type of change” (activation/deactivation) factor, resulting in two 2 × 2 × 2-mixed ANOVA designs. To further characterize the effects in the ANOVA designs, *post-hoc* comparisons were conducted using ANOVA sub-designs and pairwise comparisons. Due to an unequal distribution of participants’ sex in the groups, exploratory analyses including the additional factor “sex” (female/male) were computed to examine the influence of this variable on the reported bodily sensations related to valence and arousal.

To investigate the links between participants’ emotion-related bodily sensations, gastrointestinal-specific anxiety, and emotional awareness, Spearman’s rank correlation coefficients were calculated. In addition to our preregistered analyses (see below), ROI scores for the experience of valence and arousal in distinct parts of the body were analyzed using exploratory 2 × 2 × 2 rank-aligned ANOVA designs with the same factors as those used in the whole-body analyses. The statistical analyses were carried out using SPSS v.27.0 (IBM Corp., United States) and the ARTool package implemented in RStudio v.1.2.1335 (RStudio, PBC, Boston, MA, United States).^2^ The significance level was set to *p* < 0.05. To control for multiple testing in the correlation analyses, we applied a false-discovery rate (FDR) correction, as recommended by Benjamini and Hochberg (32), and we reported the corresponding *p*-values, denoted as *p*_*FDR*_. For all analyses of an exploratory nature, uncorrected *p*-values are reported.

## Preregistration

The hypotheses, sample size, methods, exclusion criteria, and planned analyses were preregistered before data collection and can be accessed at https://aspredicted.org/blind.php?x=hu4n7k. According to our preregistration, a sample size of 120 participants was planned. However, because of the COVID-19 outbreak and pandemic-related restrictions, the number of participants was reduced. Furthermore, in contrast to our preregistered recruitment procedure, the HC group comprised more women and less men compared to the IBD group, resulting in two samples not fully matched as initially planned. Mean age and education level of all participants have been balanced and both groups were comparable with respect to these variables. All remaining aspects of the study were carried out in accordance with the pre-registered protocol unless stated otherwise. Note that because of length restrictions, only data on emotional experience of valence and arousal are reported in this manuscript. Data on body image perception and the experience of specific emotions, as described in the same preregistration, are reported in separate articles.

## Results

### Valence: Positive and Negative Emotions

#### Whole-Body Sensations

**FIGURE 1:**
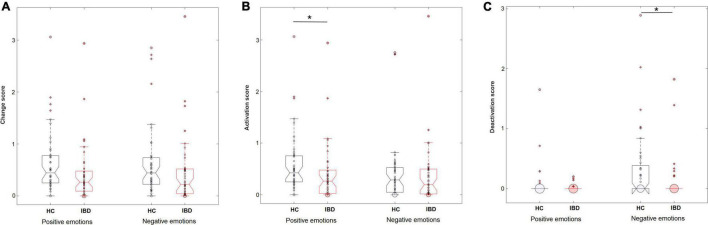
Perceived levels of overall changes in the body **(A)**, sensations of activation **(B)**, and sensations of deactivation **(C)** in the body during the experience of positive and negative emotions in HC (*n* = 44) and IBD (*n* = 41). *p < 0.05.

IBD patients reported significantly less perceived change in their bodily sensations compared to HC (main effect “group”: *F*_1_, _83_ = 6.90, *p* = 0.010, *_*p*_*η^2^ = 0.077), without a difference between positive and negative emotions (main effect “valence”: *F*_1_, _83_ = 0.23, *p* = 0.635, *_*p*_*η^2^ = 0.003; group × valence: *F*_1_, _83_ < 0.01, *p* = 0.988, *_*p*_*η^2^ < 0.0001; Figure 1). To investigate whether participants’ sex influenced these effects, we repeated the analyses with “sex” as an additional between-subjects factor. These revealed no influence of the factor “sex.” For further details see Supplementary Table 6.

Separating the changes in bodily activation and deactivation revealed that differences between the groups depended on whether the participants judged activation or deactivation for positive or negative emotions (group × valence × type of change: *F*_1_, _249_ = 6.47, *p* = 0.012, *_*p*_*η^2^ = 0.025). Decomposing the design into 2 × 2 ANOVA sub-designs revealed a significant group difference for bodily deactivation, influenced by the emotional valence (group × valence: *F*_1_, _83_ = 9.56, *p* = 0.003, *_*p*_*η^2^ = 0.103): Compared with HC, IBD patients reported a smaller difference in the perceived deactivation between positive and negative emotions (*p* = 0.005), caused by a lower deactivation associated with negative emotions (*p* = 0.015, positive emotions: *p* = 0.746; see Figure 1). For bodily activation, the groups differed depending on the emotional valence at a trend-level significance level (2 × 2 ANOVA sub-design for activation: main effect “group”: *F*_1_, _83_ = 4.84, *p* = 0.031, *_*p*_*η^2^ = 0.055; group × valence: *F*_1_, _83_ = 3.81, *p* = 0.054, *_*p*_*η^2^ = 0.044). Compared with HC, the IBD group reported a smaller difference between positive and negative emotions (*p* = 0.018). This effect was caused by a significantly lower bodily activation during positive emotions (*p* = 0.015) but not during negative emotions in the IBD group (*p* = 0.587; see Figure 1). All main and interaction effects are reported in Table 2 but are not further described because of their restricted interpretability caused by higher-order interaction effects.

**TABLE 2:** Results of the 2 × 2 × 2 rank-aligned ANOVA for whole-body valence and arousal scores.

	*F*	*df*	*p*-value	*_*p*_*η^2^
**Valence**				
Group	7.95	1/83	0.006**	0.082
Emotional valence	3.69	1/249	0.056(*)	0.002
Type of change	107.91	1/249	<0.001***	0.043
Group × Emotional valence	0.14	1/249	0.707	0.011
Group × Type of change	1.08	1/249	0.300	<0.001
Emotional valence × Type of change	18.53	1/249	<0.001***	0.073
Group × Emotional valence × Type of change	6.47	1/249	0.012*	0.025
**Arousal**				
Group	12.94	1/83	<0.001***	0.133
Arousal	4.03	1/249	0.046*	0.029
Type of change	7.01	1/249	0.009**	0.203
Group × Arousal	0.00	1/249	0.956	<0.010
Group × Type of change	2.82	1/249	0.094(*)	0.007
Arousal × Type of change	56.16	1/249	<0.001***	0.252
Group × Arousal × Type of change	7.06	1/249	0.008**	0.006

(*)Non-significant trend, *p < 0.05, **p < 0.01, ***p < 0.001.

Extending the analysis by the additional factor “sex” revealed that the group differences depending on the factors “valence” and “type of change” were not influenced by the participant’s sex (group × sex × valence × type of change: *F*_1_, _243_ = 0.05, *p* = 0.826, *_*p*_*η^2^ < 0.001; group × valence × type of change: *F*_1_, _243_ = 6.02, *p* = 0.015, *_*p*_*η^2^ = 0.024). However, independently of the assessed emotional valence, sex influenced the group differences in the reported bodily activation and deactivation (group × sex × type of change: *F*_1_, _243_ = 5.51, *p* = 0.020, *_*p*_*η^2^ = 0.022). Decomposing the design into two separate 2 × 2 × 2 ANOVA sub-designs indicated that this effect was caused by lower perceived deactivation reported by male patients with IBD compared to male healthy control participants (*p* = 0.041; Supplementary Figure 5) while there was no difference between female patients with IBD and female HC. For further details see Supplementary Table 7.

#### Exploratory Regions of Interest Analyses

Statistical analyses of the ROI scores revealed that the groups differed only in the overall change in their bodily sensations experienced in the back (main effect “group”: *F*_1_, _83_ = 7.75, *p* = 0.007, *_*p*_*η^2^ = 0.085), with IBD patients reporting less change of sensation in this area compared to HC (see Supplementary Table 2).

**FIGURE 2:**
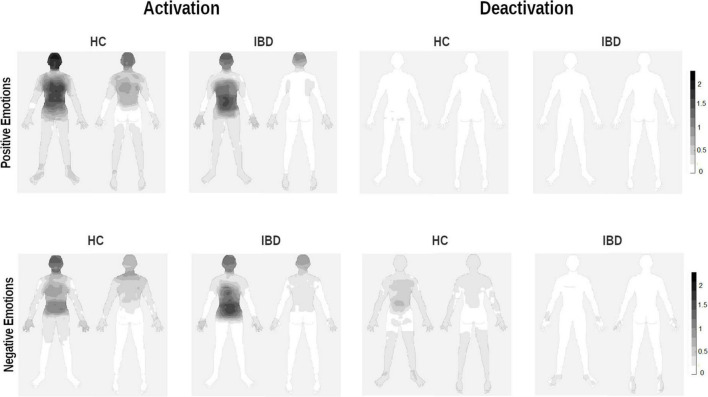
Whole-body topographies visualizing the perceived activation and deactivation for the experience of positive and negative emotions in HC participants and IBD patients (0 = “no activation/deactivation”; 4 = “very strong activation/deactivation”).

Inflammatory bowel diseases and HC participants differed in their differentiation between bodily activation and deactivation depending on whether they evaluated positive or negative emotions. This pattern was observed for the chest, legs, and back, as well as at trend-level significance levels for the head, arms, and abdomen (group × valence × type of change; see Table 3). 2 × 2 ANOVA sub-designs revealed that compared to HC, IBD patients reported smaller differences in their bodily deactivation between positive and negative emotions for the head, chest, arms, back, and abdomen (all *ps* < 0.03), resulting from a lower perceived deactivation during negative emotions (all *ps* ≤ 0.044) but not during positive emotions (all *ps* ≥ 0.890; see Figure 2). For body activation, the two groups differed significantly, with IBD patients reporting less perceived activation in the back and legs, independent of the emotional valence (2 × 2 ANOVA sub-design for activation: main effects “group”: all *ps* ≤ 0.014; group × valence: *ps* = 0.081). For further details, see Supplementary Figure 3.

**TABLE 3:** Interaction effects between the factors “Group” (IBD/HC), “Valence” (positive/negative), “Arousal” (relaxed/tensed), and “Type of change” (activation/deactivation) for all predefined ROI.

	Valence			Arousal		
	Group × Valence × Type of change			Group × Arousal × Type of change		
	*F*_1,249_	*p*	*_*p*_*η^2^	*F*_1,249_	*p*	*_*p*_*η^2^
Abdomen	3.46	0.064(*)	0.014	0.67	0.414	0.003
Head	2.76	0.098(*)	0.011	11.56	<0.001***	0.044
Chest	6.37	0.012*	0.025	2.46	0.118	0.009
Arms	3.04	0.082(*)	0.012	7.09	0.008**	0.028
Legs	6.31	0.013*	0.025	1.88	0.171	0.008
Back	3.92	0.049*	0.016	2.18	0.141	0.009

(*)Non-significant trend, *p < 0.05, **p < 0.01, ***p < 0.001; for details on the other experimental factors, see Supplementary Table 3.

### Arousal: Relaxation and Tension

#### Whole-Body Sensations

**FIGURE 3:**
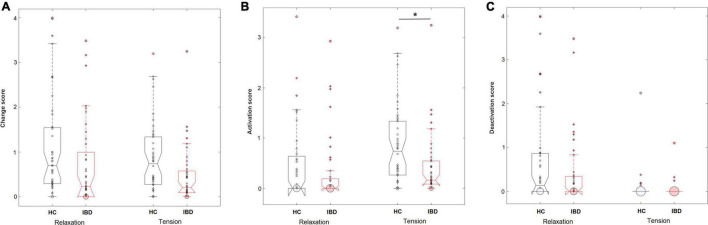
Perceived levels of overall changes in the body **(A)**, sensations of activation **(B)**, and sensations of deactivation **(C)** in the body during the experience of relaxation and tension in HC (*n* = 44) and IBD (*n* = 41). *p < 0.05.

IBD patients reported less perceived change in their bodily sensations related to the experience of arousal (main effect “group”: *F*_1_, _83_ = 10.20, *p* = 0.002, *_*p*_*η^2^ = 0.109) without a difference between the experiences of relaxation and tension (group × arousal: *F*_1_, _83_ = 0.52, *p* = 0.474, *_*p*_*η^2^ = 0.006; main effect “arousal”: *F*_1_, _83_ = 0.52, *p* = 0.474, *_*p*_*η^2^ = 0.006). The medians and data ranges for these conditions are shown in Figure 3. Extending the ANOVA design by the additional between-subjects factor “sex” revealed no influence of the participants’ sex on the evaluation of bodily sensation related to arousal. For further details see Supplementary Table 6.

**FIGURE 4:**
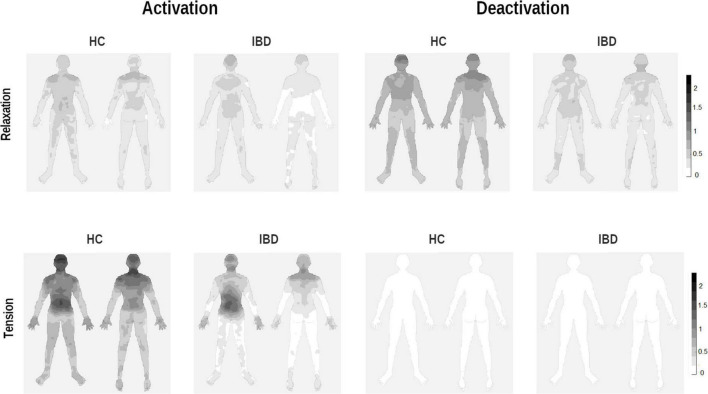
Whole-body topographies visualizing perceived activation and deactivation for the experience of relaxation and tension in HC participants and IBD patients (0 = “no activation/deactivation”; 4 = “very strong activation/deactivation”).

When separating the effects of bodily activation and deactivation, analyses showed a significant effect of the type of change on the group difference for relaxation and tension (group × arousal × type of change: *F*_1_, _249_ = 7.06, *p* = 0.008, *_*p*_*η^2^ = 0.028). 2 × 2 ANOVA sub-designs revealed a significant group difference for the perceived bodily deactivation, depending on whether participants evaluated relaxation or tension (group × arousal: *F*_1_, _83_ = 6.05, *p* = 0.016, *_*p*_*η^2^ = 0.068). Compared to HC, IBD patients reported a smaller difference in the experienced bodily deactivation during relaxation and tension (*p* = 0.001). For bodily activation, the two groups differed significantly depending on whether relaxation or tension was evaluated (2 × 2 ANOVA sub-design for activation: group × arousal: *F*_1_, _83_ = 9.39, *p* = 0.003, *_*p*_*η^2^ = 0.102): Compared to HC, the IBD group exhibited a smaller difference in their experienced bodily activation for relaxation and tension, resulting from a significantly lower perceived activation during tension (*p* = 0.002; Figure 4) but not during relaxation (*p* = 0.152). For further details, see Table 2.

Exploratory analyses including the additional factor “sex” revealed that differences between the groups depending on arousal and the type of change were influenced by the participant’s sex (group × sex × arousal × type of change interaction effect: *F*_1_, _243_ = 12.41, *p* < 0.001, *_*p*_*η^2^ = 0.049). Decomposing the design into two separate 2 × 2 × 2 ANOVA sub-designs for activation and deactivation scores revealed significant 3-way interactions in both analyses (group × sex × arousal: bodily activation: *F*_1_, _81_ = 6.08, *p* = 0.016, *_*p*_*η^2^ = 0.069; bodily deactivation: *F*_1_, _81_ = 12.37, *p* < 0.001, *_*p*_*η^2^ = 0.133) which, however, suggest a differential pattern of influence of participant’s sex on the reported bodily activation and deactivation scores. For further details see Supplementary Table 7.

Differences in the reported bodily activation were caused by lower scores for the experience of tension among male IBD patients compared to male healthy control participants (*p* = 0.002; Supplementary Figure 6), without differences for females with and without IBD (*p* = 0.713). For bodily deactivation, male patients with IBD reported significantly lower deactivation scores for the experience of relaxation compared to healthy male participants (*p* = 0.046; Supplementary Figure 7), while no differences were found for the experience of tension (*p* = 0.408). Females did not differ in the reported deactivation scores for relaxation or tension (*p*s > 0.812).

#### Exploratory Regions of Interest Analyses

Inflammatory bowel diseases patients reported less perceived change in their bodily sensations across all ROIs (all main effects “group”: *ps* < 0.044), except for the abdomen (*p* = 0.987; see Supplementary Table 2).

For the head and the arms, the statistical analysis results showed that the differences between the two groups were influenced by whether the participants judged changes to be linked to relaxation or tension and activation or deactivation (group × arousal × type of change, see Table 3). 2 × 2 ANOVA sub-designs showed that the group difference in bodily deactivation and activation was influenced by whether relaxation or tension was evaluated (group × arousal for deactivation: *ps* ≤ 0.003; activation: *ps* ≤ 0.011). Compared to HC, IBD patients exhibited a significantly smaller difference in their perceived bodily deactivation during relaxation and tension (all *ps* ≤ 0.005), caused by less perceived deactivation for relaxation in the head and arms in IBD (*ps* ≤ 0.097; see Figure 4). For bodily activation, the difference between relaxation and tension was significantly smaller in the IBD group than in the HC group (*p*s ≤ 0.001): IBD patients reported significantly lower levels of activation during tension than those in the HC group (*ps* ≤ 0.009). For further details, see Supplementary Figure 4.

## Correlations Between Emotion-Associated Bodily Sensations and Interoceptive Sensibility or Gastrointestinal-Specific Anxiety

### Emotional Awareness

While changes in bodily sensations for valence and arousal were stronger in participants reporting higher levels of emotional awareness, there were no differences between the groups in these relationships. For further details, see Supplementary Table 4.

#### Valence

In the differentiation between bodily activation and deactivation, higher emotional awareness was linked to greater bodily activation for the experience of positive emotions only in the IBD group (IBD: *r*_*s*_ = 0.462, *p* = 0.002, *p_*FDR*_* = 0.004; HC: *r*_*s*_ = 0.116, *p* = 0.454, *p_*FDR*_* = 0.454, comparison between groups: *Z* = 1.702, *p* = 0.044). In contrast, higher emotional awareness was linked to stronger bodily activations during negative emotions to a comparable extent in both groups (IBD: *r*_*s*_ = 0.381, *p* = 0.014, *p_*FDR*_* = 0.028; HC: *r*_*s*_ = 0.304, *p* = 0.045, *p_*FDR*_* = 0.090, comparison between groups *Z* = 0.388, *p* = 0.349).

#### Arousal

Higher emotional awareness was associated with higher levels of bodily activation during tension in the IBD group at trend-level significance. The two groups did not differ in the strength of this association (IBD: *r*_*s*_ = 0.339, *p* = 0.030, *p_*FDR*_* = 0.060; HC: *r*_*s*_ = 0.284, *p* = 0.062, *p_*FDR*_* = 0.124; *Z* = 0.271, *p* = 0.393). No significant associations were found for relaxation (IBD: *r*_*s*_ = 0.188, *p* = 0.238, *p_*FDR*_* = 0.238; HC: *r*_*s*_ = 0.271, *p* = 0.076, *p_*FDR*_* = 0.152; comparison between groups: *Z* = −0.389, *p* = 0.348).

There were no significant associations between emotional awareness and perceived bodily deactivation for either valence or arousal for either group (all *p_*FDR*_* ≥ 0.426).

### Gastrointestinal-Specific Anxiety

For the IBD group, the correlation analysis results revealed no significant associations between GSA and patients’ perceived bodily sensations related to the experience of valence and arousal (all *p_*FDR*_* ≥ 0.252).

### Anxiety and Depression Symptoms

Although all IBD patients met the criteria for clinical remission, they reported higher levels of anxiety and—at least on a descriptive level—depression than the HC participants (see Table 1). Therefore, we performed additional correlation analyses to explore whether symptoms of anxiety and depression are related to the experience of valence and arousal in the body. In the IBD group, greater anxiety and depression were associated with higher bodily activation during negative emotions (all *ps* < 0.001) and tension (all *ps* ≤ 0.011) and higher levels of deactivation when feeling relaxed (all *ps* ≤ 0.019). For further details, see Supplementary Table 5.

## Discussion

How bodily sensations are perceived and evaluated in the context of emotional experiences is essential to the way people perceive emotions in everyday life and—in the case of IBD—ultimately how these influence patient’s psychological wellbeing. Since studies on emotional experience in IBD are sparse, the main objective of this study was to investigate the potential alterations in emotion-related bodily sensations and their link to interoceptive sensibility and gastrointestinal-specific anxiety (GSA) in remitted IBD patients compared with healthy individuals. Based on the dimensional model of emotion (14), we investigated how the two dimensions of valence and arousal are experienced in an embodied way by examining perceived bodily activation and deactivation during positive and negative emotions, relaxation, and tension.

Compared to healthy individuals, patients with IBD reported less intense bodily changes associated with the experience of valence and arousal. Moreover, IBD patients varied differently in their perceived sensations of the opposite poles of these dimensions, that is, positive and negative emotions for valence and relaxation and tension for arousal, respectively. Our findings support the need for fine-grained analysis to capture the complex changes in emotion-related bodily sensations, since group differences depend on whether participants evaluate perceived activation or deactivation in different areas of the body. For IBD patients, the patterns of bodily activation and deactivation reported for positive and negative emotions, as well as for relaxation and tension, were more similar than these observed among healthy individuals. The reduced bodily activation during negative and highly arousing emotional states reported for the IBD group might suggest less intense and thereby less distressing emotional experience. However, lower levels of activation linked to positive emotions and decreased deactivation during relaxation might point to detrimental alterations in these domains of emotional experience, which might be of particular importance for patients’ wellbeing. Our results of diminished emotion-related bodily sensations in IBD indicate a more detached experience of emotions, suggesting an emotional avoidance mechanism in this patient group. While patients might perceive this strategy as helpful to avoid a confrontation with overwhelming feelings and stress (33), emotional avoidance may contribute to the maintenance of anxiety and depressive symptoms in the long term (34–36). Finally, exploratory analyses suggest that differences between patients with IBD and healthy control participants differ between men and women, particularly when evaluating bodily sensations related to the experience of arousal with stronger effects in men compared to women.

### Experience of Valence in the Body

Overall, IBD patients exhibited significantly less perceived change in their emotion-related bodily sensations. Despite the higher levels of subjectively perceived emotional awareness observed in the IBD group, when asked to identify changes in their bodies, patients reported less intense experiences of bodily activation and deactivation compared to healthy individuals. This finding may indicate that patients with IBD are less able to differentiate between the bodily sensations linked to specific emotion experiences. In line with this interpretation, several studies have demonstrated higher levels of alexithymia in IBD, that is the difficulty to name and differentiate between different emotions (37). Moreover, recent evidence have indicated impairments in patients’ ability to recognize their own feelings and those of others (38). Taken together, these findings suggest that IBD symptoms detrimentally affect patients’ capacity to discriminate between different emotions based on their bodily sensations.

We observed decreased body deactivation during negative emotional states in IBD for several body areas, including the one most affected by IBD symptoms, that is, the abdomen, as well as other less directly disease-related body regions. Thus, altered experience of negative emotions in the body is not limited only to body regions associated with aberrant physiological feedback in IBD, such as gastric sensations, for example, as initially expected. In contrast to Vianna et al. (11), who suggested that emotional dysfunctions occur only for acute IBD patients, we found differences in how emotions are experienced among remitted IBD patients. More precisely, our findings of less activation during positive emotional states suggest decreased sensitivity to positive emotional content among patients with IBD. Previous studies have suggested functional alterations in brain regions associated with the processing of affective information (10) and visceral signals in IBD patients (39, 40). Therefore, impaired integration of affective and visceral signals in IBD patients could result in less intense experience of positive emotions, leading to more depressive symptoms (41), which can in turn affect the course of the disease (9). As these findings indicate persistent alterations in the experience of positive emotions, even in the remitted stage of the disorder, one might speculate whether it might be useful to coach patients to notice sensations of activation in the body specifically related to positive emotions. Mind-body interventions, such as mindfulness-based stress reduction (MBSR) (42), emphasize the focus on bodily sensations and the appraisal of those sensations as crucial to an individual’s coping with stress and emotions (23, 43, 44). Such interventions are believed to enhance an individual’s sensitivity to body-related emotional information and to encourage the implementation of skillful strategies for coping with difficult emotions. In a segment of IBD patients, that is, those who exhibit difficulties in coping with emotional stress, short interoceptively focused interventions might be useful by coaching patients to localize changes in their visceral sensations and learn to perceive emotion-specific changes in their bodily sensations, focusing on particular body areas (45, 46). By learning to accept emotions and the corresponding physiological reactions of the body, patients could learn to observe these sensations without immediately feeling overwhelmed and trying to avoid these using distraction strategies [for a review of mind-body interventions in IBD see: (47, 48)]. By modifying how patients attend to the interoceptive signals elicited by emotional events and providing them with adaptive emotion regulation skills, such interventions can improve patients’ flexibility in emotional experience and prevent an overgeneralization of avoidance strategies of disease related bodily sensations to those associated with emotions (49, 50). Given the bidirectional relationship between emotion perception and psychopathological symptoms, it should be noted that as our results are only of correlational nature, these clinical implications are only speculative and need further investigation. Finally, exploratory analyses indicated that male patients with IBD reported less perceived bodily deactivation compared to male healthy individuals independently whether they judged bodily sensations associated with positive or negative valence. While this differential effect did not restrict the findings observed for the sensations of positive and negative valence between individuals with and without IBD, it emphasizes the need to take the participant’s sex into account when studying alterations in bodily sensations in IBD.

### Experience of Arousal in the Body

With respect to arousal, IBD patients perceived less bodily activation during tension than HC, while noticing smaller differences in deactivation experienced during relaxation and tension. In contrast to valence, this pattern was reported only for body areas not directly related to IBD symptomatology. Despite patients’ lower levels of bodily activation when feeling tense, our results indicate that patients with IBD perceive less bodily deactivation during relaxation and therefore less successfully reduce perceived stress and tension (51, 52). Hence, IBD patients may benefit from practicing relaxation techniques, such as autogenic training or progressive muscle relaxation, in order to better differentiate between bodily sensations caused by tension and relaxation (51, 53). Previous studies have shown the positive effects of such interventions on stress reduction in psychosomatic disorders, suggesting potential benefits for IBD patients’ mental and physical wellbeing (54).

Our findings of significantly less intense perceived bodily sensations associated with valence and arousal may indicate the use of emotional avoidance as an emotion regulation strategy among IBD patients. A more detached bodily experience of emotions may allow patients to avoid confronting overly intense physiological sensations and thereby reduce psychological distress. Consistent with this interpretation, a recent study by Banovic et al. (55) found stronger emotional avoidance and emotional suppression among Crohn’s disease patients than among healthy controls (55). Moreover, previous studies on attachment and mentalizing in IBD revealed greater avoidance tendencies in this clinical population (38, 56). Yet, an avoidant attachment style has been associated with cognitive distancing from emotions and our findings suggest a detachment from the corresponding emotion-related bodily sensations as well. These defensive strategies, however, might further impede patients’ mentalization abilities, leading to interpersonal problems (38). However, another explanation for our results could be the use of distraction as a regulatory strategy. Contemporary findings point to the benefits of different emotion regulation mechanisms depending on the intensity of the emotional state (57). While cognitive reappraisal is thought to be more adaptive in the case of low negative emotional intensities, distraction is preferable when individuals face emotions of high intensity (58). As high emotional intensity usually results in greater activation of the autonomic nervous system, it is conceivable that individuals who are more sensitive to these physiological changes may prefer to use distraction to cope with highly arousing emotions (49). Future studies should investigate whether altered processing of bodily sensations is linked to different emotion regulation strategies and whether these are differentially advantageous for coping with disease-related psychological distress and IBD symptoms. Such research might improve our understanding of the mechanisms underlying the interplay between emotion regulation and the experience of emotions in the body and thereby facilitate the development of personalized psychotherapeutic interventions for IBD. Finally, the patterns related to the experience of arousal differed between female and male participants. While females with and without IBD did not differ significantly, compared to healthy male participants, males with an IBD diagnosis exhibited decreased sensations of bodily deactivation for the experience of relaxation. On the other hand, male patients with IBD reported lower levels of bodily activation during tension, which suggests that patients might show a stronger detachment from their bodily sensations during stress, while experiencing greater difficulties to relax and thus, in coping with sustained stress-related sensations. While recent findings suggest that there are no clear sex differences in the subjective experience of emotions in the body (59) but rather only in the expression of those (60), our findings indicate that men with IBD show attenuated emotion-related sensations, suggesting a stronger emotional avoidance.

### Correlations With Interoceptive Sensibility, Gastrointestinal-Specific Anxiety, and Psychological Distress

Inflammatory bowel diseases patients reporting higher levels of emotional awareness, that is, a feature of one’s interoceptive sensibility, exhibit increased bodily sensations during positive and negative emotions, as well as tension (61). The effects of interoception on emotional experience have been discussed repeatedly in the literature, suggesting that interoceptively aware individuals report higher emotional intensity and arousal (62, 63). IBD patients exhibiting higher emotional awareness also report higher levels of bodily activation during positive emotions. Although positive valence is associated with less perceived bodily activation in IBD patients, greater awareness of the connection between physiological sensations and emotions can positively affect the experience of positive emotions. One possible explanation for this effect might be that body- and emotion perception share common neural underpinnings (64), playing crucial roles in the integration of interoceptive and emotional cues (65, 66). Critchley et al. (67) demonstrated that representations of internal bodily sensations in the insula are crucial for the conscious experience of emotions. This suggests that somato-visceral information influences emotion perception in a bottom-up way, indicating that individuals who are more aware of their bodily signals exhibit higher emotional awareness and thus perceive more intense emotions. IBD has been associated with altered activation of the insula, characterized by hyperactivation during visceral pain and stress perception (68, 69). This enhanced insular reactivity might constitute the neural correlate of an increased perception of visceral sensations in IBD and a stronger experience of emotion-related sensations, particularly in those patients who report to be more aware of their body signals.

Our results did not reveal any associations between gastrointestinal-specific anxiety (GSA) and emotion experience in the body. In contrast to GSA, which solely reflects patients’ disease-related cognitions, more pronounced general anxiety and depression symptoms were linked to greater bodily activation during positive and negative emotions. This finding is in line with previous evidence that suggests that highly anxious individuals experience emotions in a more intense and threatening way (70). As acute disease activity is normally linked to higher levels of anxiety and depression, this result might indicate that among acute IBD patients, increased emotion-related bodily sensations linked to psychological distress might result in exaggerated emotion perception having detrimental effects on patients’ stress levels, at least for those patients with psychiatric co-morbidities (71).

Given these new findings regarding the role of bodily sensations related to the experience of valence and arousal in IBD, the implementation of interoceptively focused techniques to improve patients’ emotion experience through greater bodily awareness as a supplementary part of patients’ treatment might be of particular importance. Our results raise the possibility that psychological interventions aimed at the improvement of emotion perception in IBD could provide patients with useful skills for engaging with their emotions. As impairments in emotion perception have been shown to be part of the link between disease severity and depression, such supplementary interventions could improve patients’ abilities to cope with their disease symptoms and thus, potentially having positive effects on their psychological wellbeing and quality of life.

## Limitations

Some limitations of the present study have to be addressed. First, in contrast to our previously preregistered matching procedure, more female than male healthy individuals were included in the HC group, resulting in an unequal sex distribution between both groups. Second, as we did not include a clinical control group (e.g., irritable bowel syndrome, IBS) and did not collect data on IBS symptoms in the IBD group, we cannot conclude that our findings are limited only to IBD or that they may represent a transdiagnostic feature across several gastrointestinal disorders or even across somatic and mental disorders. Further, we did not directly induce any emotional states but rather asked participants to indicate changes in their bodily sensations based on what they typically perceive when experiencing the presented emotional state. Thus, the participants’ judgments resulted from their recollections of bodily sensations during emotional states in the past. This recall process might be biased by one’s current mood or by recalling only recent situations. Therefore, future studies need to control for these effects by asking participants to indicate their bodily sensations during direct emotion induction. Furthermore, interoception-related features were assessed only *via* self-report measures, which often do not correspond to participant’s actual interoceptive abilities (72, 73). Moreover, no data on perceived stress and quality of life was collected and thus, future studies are needed to examine the complex interplay of emotion and stress perception as well as their links to quality of life in IBD. Finally, we cannot draw conclusions regarding the direction of causality of any of the observed associations because of the cross-sectional design of the study.

## Conclusion

The results of this study indicate that IBD is associated with emotional alterations, characterized by attenuated experiences of emotion-related bodily sensations. The observed stronger detachment from one’s bodily sensations might be a regulatory mechanism emerging from the individual’s attempt to avoid distressing bodily sensations, associated with IBD symptoms and pain, which affects the perception and evaluation of emotion-related bodily sensations due to overgeneralization. The links between emotion-related bodily sensations and anxiety and depressive symptoms suggest that altered emotion perception might contribute to the development and maintenance of psychopathological symptoms during the disease course. However, further studies are needed to investigate whether there is indeed a causal relationship between these domains of functioning and how these affect patients’ quality of life.

## Data Availability Statement

The raw data supporting the conclusions of this article will be made available by the authors, without undue reservation.

## Ethics Statement

The studies involving human participants were reviewed and approved by the Ethics Committee of the Medical Faculty Mannheim, Heidelberg University. The patients/participants provided their written informed consent to participate in this study.

## Author Contributions

KA, WR, and SL designed the study and wrote the protocol. KA, TL, and WR recruited the sample. KA performed the data collection, conducted all statistical analyses, and wrote the first draft of the manuscript. SL, WR, TL, RB-B, and NK provided substantive and conceptual feedback on all drafts. All authors contributed to and have approved the final manuscript.

## Conflict of Interest

The authors declare that the research was conducted in the absence of any commercial or financial relationships that could be construed as a potential conflict of interest.

## Publisher’s Note

All claims expressed in this article are solely those of the authors and do not necessarily represent those of their affiliated organizations, or those of the publisher, the editors and the reviewers. Any product that may be evaluated in this article, or claim that may be made by its manufacturer, is not guaranteed or endorsed by the publisher.

## Footnotes

1
www.neurobs.com

2
http://www.rstudio.com/
