# Supplementary material for: Is It a Gut Feeling? Bodily Sensations Associated With the Experience of Valence and Arousal in Patients With Inflammatory Bowel Disease
Source: Front Psychiatry. 2022 Apr 22;13:833423. doi: 10.3389/fpsyt.2022.833423 (PMC9072626; doi:10.3389/fpsyt.2022.833423)
Supplement: Supplementary file 1 [file Data_Sheet_1.docx]

Supplementary Material

**Is it a gut feeling? Bodily sensations associated with the experience of valence and arousal in patients with inflammatory bowel disease**

**Table S1.** Treatment medication, history of other somatic diseases, previous surgeries, and history of extraintestinal manifestations in the IBD group (N = 41)

| **IBD medication** | **N** |
| --- | --- |
| Biologics | 40 |
| Mesalazine | 1 |
| **Other somatic condition** |  |
| Pulmonary embolism | 2 |
| Pancreatitis | 2 |
| Diabetes | 1 |
| Chronic cystitis | 1 |
| Thrombocythemia | 1 |
| Asthma bronchiale | 1 |
| Hypothyoroidism | 2 |
| **IBD-related surgeries** |  |
| No surgeries | 19 |
| Ileal resection | 9 |
| Fistula removal | 8 |
| Abscess | 5 |
| **Extraintestinal manifestations** |  |
| Entheropathic arthritis | 6 |
| Dermatitis | 4 |

**Interoceptive sensibility: Multidimensional Assessment of Interoceptive Awareness (MAIA)**

The 32 items self-report questionnaire measures subjective interoceptive abilities as a complex construct involving attentional and emotional processes. MAIA provides a multidimensional profile of body awareness including following eight subscales: *Noticing*, *Not-Distracting*, *Not-Worrying*, *Attention Regulation*, *Emotional Awareness*, *Self-Regulation*, *Body Listening* and *Trusting*. With respect to interoceptive sensibility, the Emotional awareness subscale assesses individual’s awareness of the connection between bodily sensations and emotional states (item example: “I notice how my body changes when I am angry”) (Mehling et al., 2012). The scores of all subscales range between 0 and 5 with higher scores indicating greater interoceptive sensibility.

Mehling, W. E., Price, C., Daubenmier, J. J., Acree, M., Bartmess, E., & Stewart, A. (2012). The Multidimensional Assessment of Interoceptive Awareness (MAIA). *PLoS One, 7*(11), e48230. <https://doi.org/10.1371/journal.pone.0048230>

**
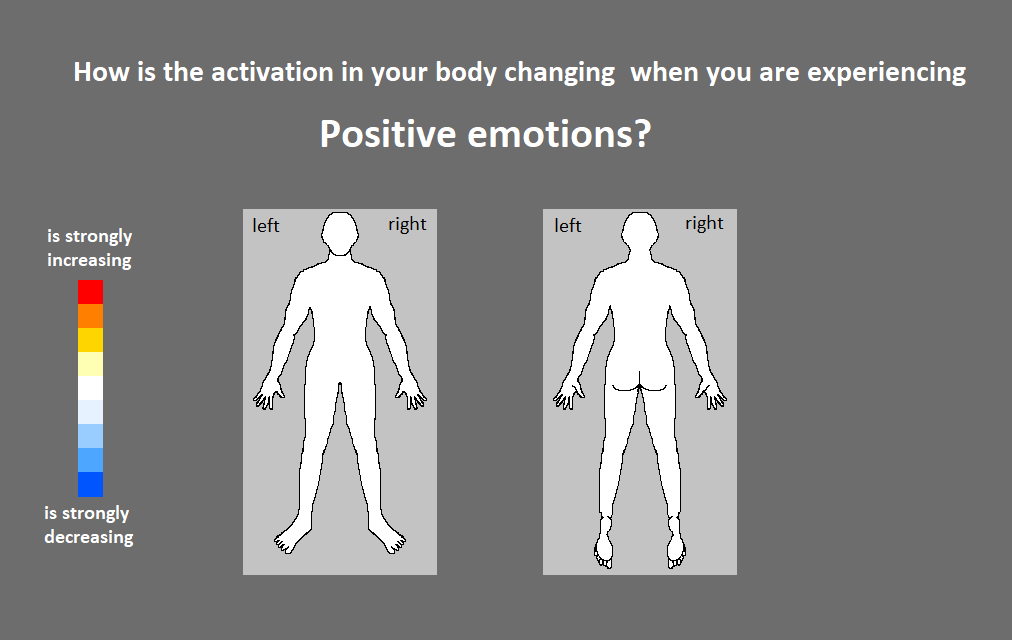
**

**Figure S1.** Experimental task overview. Participants were asked to color the body areas they feel becoming more activated or deactivated by selecting a color from a 9-point color bar ranging from blue (- 4 = “very strong deactivation”) to red (4 = “very strong activation”).


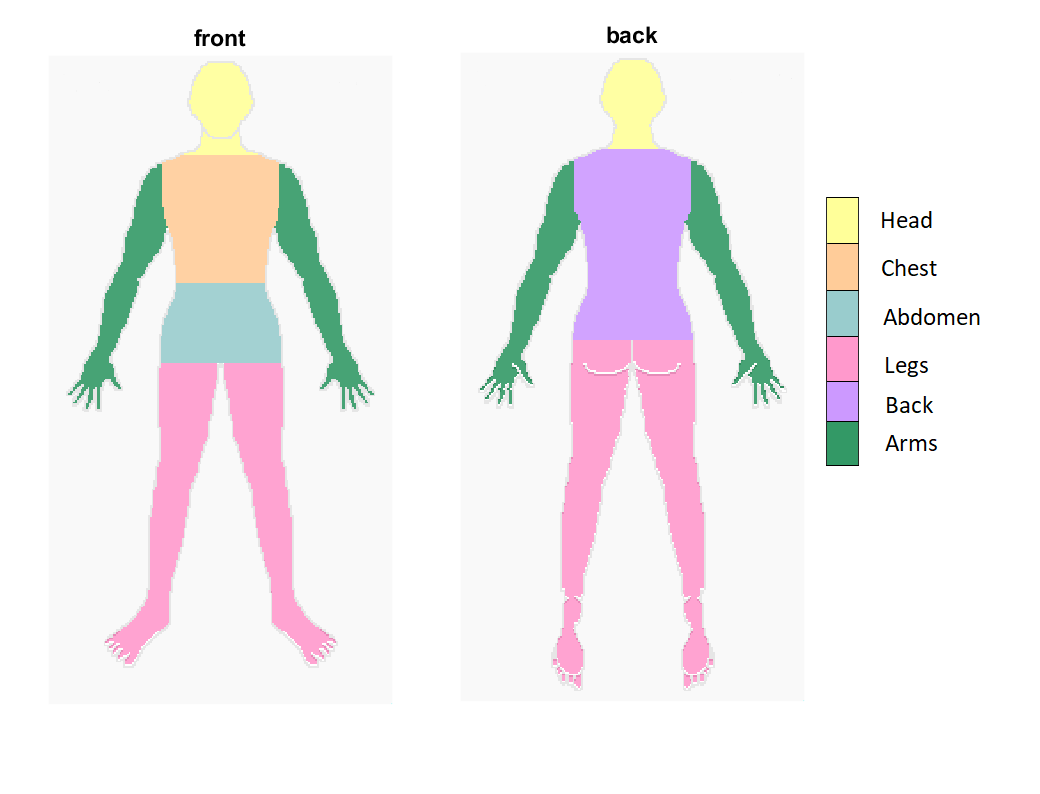


**Figure S2.** Pre-defined regions of interest (ROI). ROI included the head, chest, arms, abdomen, legs, and the back of the presented body template.

**Table S2.** Results of 2x2 mixed-effects ANOVA designs with factors “group” (IBD/HC) and the additional factor “valence” (positive/negative emotions) or “arousal” (relaxation/tension) on the overall perceived changes in the body for ROIs.

|  | *Dimension: Valence* | | | *Dimension: Arousal* | | |
| --- | --- | --- | --- | --- | --- | --- |
|  | *F* _1,83_ | *p*-value | | *F* _1,83_ | *p*-value | |
| **Abdomen** |  |  |  |  |  |  |
| Group | 0.29 | .595 |  | 0.00 | .987 |  |
| dimension | 0.54 | .464 |  | 0.55 | .458 |  |
| Group*dimension | 0.15 | .697 |  | 0.57 | .451 |  |
| **Head** |  |  |  |  |  |  |
| Group | **2.97** | **.089** | **(*)** | **4.17** | **.044** | ***** |
| dimension | 3.53 | .064 | (*) | 0.05 | .821 |  |
| Group*dimension | 2.27 | .135 |  | 0.03 | .872 |  |
| **Chest** |  |  |  |  |  |  |
| Group | **3.36** | **.069** | **(*)** | **5.99** | **.016** | ***** |
| dimension | 2.55 | .114 |  | **7.06** | **.009** | ****** |
| Group*dimension | 1.08 | .301 |  | 2.35 | .129 |  |
| **Arms** |  |  |  |  |  |  |
| Group | **3.61** | **.061** | **(*)** | **8.69** | **.004** | ****** |
| dimension | 1.46 | .230 |  | **8.83** | **.004** | ****** |
| Group*dimension | 0.53 | .470 |  | 3.43 | .068 |  |
| **Legs** |  |  |  |  |  |  |
| Group | **3.34** | **.071** | **(*)** | **4.70** | **.033** | ***** |
| dimension | 0.04 | .847 |  | 2.89 | .093 | (*) |
| Group*dimension | 0.48 | .492 |  | 1.36 | .248 |  |
| **Back** |  |  |  |  |  |  |
| Group | **7.75** | **.007** | ****** | **8.53** | **.005** | ****** |
| dimension | 0.35 | .554 |  | **6.23** | **.015** | ***** |
| Group*dimension | 0.65 | .424 |  | 1.40 | .241 |  |

Notes: statistically significant effects including the factor ‘group’ are marked in bold.

(*) *p* < .10, * *p* < .05, ** *p* < .01

**Table S3.** Results of 2x2x2 mixed-effects ANOVA designs with factors “group” (IBD/HC), type of change (activation/deactivation) and the additional factor “valence” (positive/negative emotions) or “arousal” (relaxation/tension) on the perceived changes in the body for the ROIs

|  | *Dimension: Valence* | | | | *Dimension: Arousal* | | | |
| --- | --- | --- | --- | --- | --- | --- | --- | --- |
|  | *F* | *df* | *p*-value | | *F* | *df* | *p-*value | |
| **Abdomen** |  |  |  |  |  |  |  |  |
| **Group** | **3.07** | **1/83** | **.084** | **(*)** | 0.30 | 1/83 | .582 |  |
| Dimension | 1.12 | 1/249 | .292 |  | 9.62 | 1/249 | .002 | ** |
| Type of change | 76.79 | 1/249 | < .001 | *** | 28.16 | 1/249 | < .001 | *** |
| **Group*Dimension** | **5.88** | **1/249** | **.016** | ***** | 0.16 | 1/249 | .691 |  |
| **Group*Type of change** | **5.94** | **1/249** | **.016** | ***** | 1.18 | 1/249 | .279 |  |
| Dimension*Type of change | 2.28 | 1/249 | .132 |  | 36.69 | 1/249 | < .001 | *** |
| **Group*Dimension*Type of change** | **3.46** | **1/249** | **.064** | **(*)** | .67 | 1/249 | .414 |  |
| **Head** |  |  |  |  |  |  |  |  |
| **Group** | 2.10 | 1/83 | .151 |  | **8.69** | **1/83** | **.004** | ****** |
| Dimension | 13.98 | 1/249 | < .001 | *** | 8.39 | 1/249 | .004 | ** |
| Type of change | 254.50 | 1/249 | < .001 | *** | 2.82 | 1/249 | .094 | (*) |
| **Group*Dimension** | 0.04 | 1/249 | .839 |  | 3.24 | **1/249** | **.073** | **(*)** |
| **Group*Type of change** | **13.69** | **1/249** | **< .001** | ******* | **3.56** | **1/249** | **.061** | **(*)** |
| Dimension*Type of change | 5.10 | 1/249 | .025 | * | 52.04 | 1/249 | < .001 | *** |
| **Group*Dimension*Type of change** | **2.76** | **1/249** | **.098** | **(*)** | **11.56** | **1/249** | **< .001** | ******* |
| **Chest** |  |  |  |  |  |  |  |  |
| **Group** | **3.26** | **1/83** | **.075** | **(*)** | **4.72** | **1/83** | **.033** | ***** |
| Dimension | 16.71 | 1/249 | < .001 | *** | 5.17 | 1/249 | .024 | ***** |
| Type of change | 129.67 | 1/249 | < .001 | *** | 32.25 | 1/249 | < .001 | *** |
| Group*Dimension | 2.68 | 1/249 | .103 |  | 0.08 | 1/249 | .779 |  |
| **Group*Type of change** | 1.57 | 1/249 | .211 |  | **3.27** | **1/249** | **.071** | **(*)** |
| Dimension*Type of change | 9.10 | 1/249 | .003 | ** | 39.02 | 1/249 | < .001 | *** |
| **Group*Dimension*Type of change** | **6.37** | **1/249** | **.012** | ***** | 2.46 | 1/249 | .118 |  |
| **Arms** |  |  |  |  |  |  |  |  |
| **Group** | **2.87** | **1/83** | **.094** | **(*)** | **8.61** | **1/83** | **.004** | ****** |
| Dimension | 0.50 | 1/249 | .479 |  | 4.72 | 1/249 | .031 | * |
| Type of change | 10.34 | 1/249 | .001 | ** | 0.043 | 1/249 | .836 |  |
| Group*Dimension | 0.86 | 1/249 | .354 |  | .432 | 1/249 | .511 |  |
| Group*Type of change | 0.52 | 1/249 | .472 |  | 2.05 | 1/249 | .154 |  |
| Dimension*Type of change | 1.52 | 1/249 | .218 |  | 38.34 | 1/249 | < .001 | *** |
| **Group*Dimension*Type of change** | **3.04** | **1/249** | **.082** | **(*)** | **7.09** | **1/249** | **.008** | ****** |
| **Legs** |  |  |  |  |  |  |  |  |
| **Group** | **9.72** | **1/83** | **.002** | ****** | 1.52 | 1/83 | .221 |  |
| Dimension | 0.14 | 1/249 | .710 |  | 7.36 | 1/249 | .007 | ****** |
| Type of change | 1.05 | 1/249 | .308 |  | 2.17 | 1/249 | .142 |  |
| **Group*Dimension** | 0.08 | 1/249 | .777 |  | 0.77 | 1/249 | .380 |  |
| Group*Type of change | 0.70 | 1/249 | .403 |  | 2.39 | 1/249 | .123 |  |
| Dimension*Type of change | 9.41 | 1/249 | .002 | ** | 6.47 | 1/249 | .012 | * |
| **Group*Dimension*Type of change** | **6.31** | **1/249** | **.013** | ***** | 1.88 | 1/249 | .171 |  |
| **Back** |  |  |  |  |  |  |  |  |
| **Group** | **16.57** | **1/83** | **< .001** | ******* | **6.11** | **1/83** | **.015** | ***** |
| Dimension | 1.92 | 1/249 | .167 |  | 3.88 | 1/249 | .050 | * |
| Type of change | 63.01 | 1/249 | < .001 | *** | 13.15 | 1/249 | < .001 | *** |
| Group*Dimension | 1.52 | 1/249 | .219 |  | 0.75 | 1/249 | .386 |  |
| **Group*Type of change** | **19.69** | **1/249** | **< .001** | ******* | **3.85** | **1/249** | **.051** | **(*)** |
| Dimension*Type of change | 1.56 | 1/249 | .212 |  | 48.85 | 1/249 | < .001 | *** |
| **Group*Dimension*Type of change** | **3.92** | **1/249** | **.049** | ***** | 2.17 | 1/249 | .141 |  |

Notes: Main and interaction effects including the factor ‘group’ are marked in bold. (*) *p* < .10, * *p* < .05, ** *p* < .01, *** *p* < .001

**
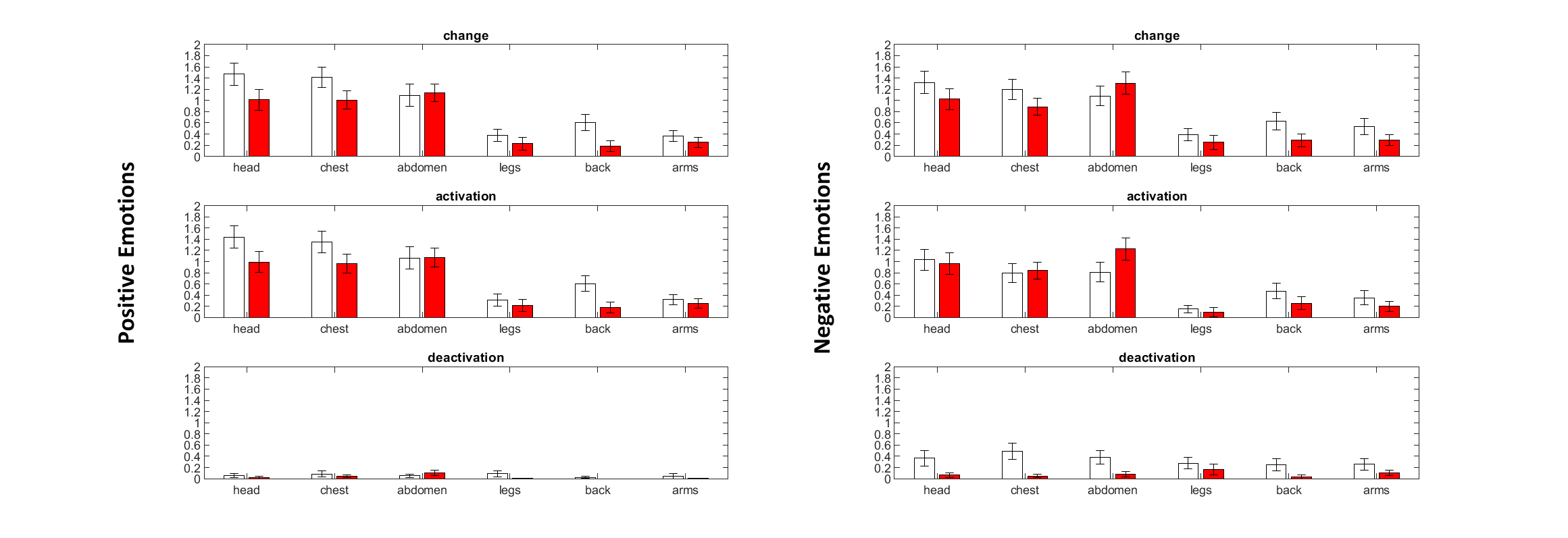
**

**Figure S3**. Means and standard deviations of the reported overall perceived bodily changes, activation, and deactivation in all pre-defined regions of interest for positive emotions (left plot) and negative emotions (right plot). Red bars indicate IBD group, white bars indicate HC group.


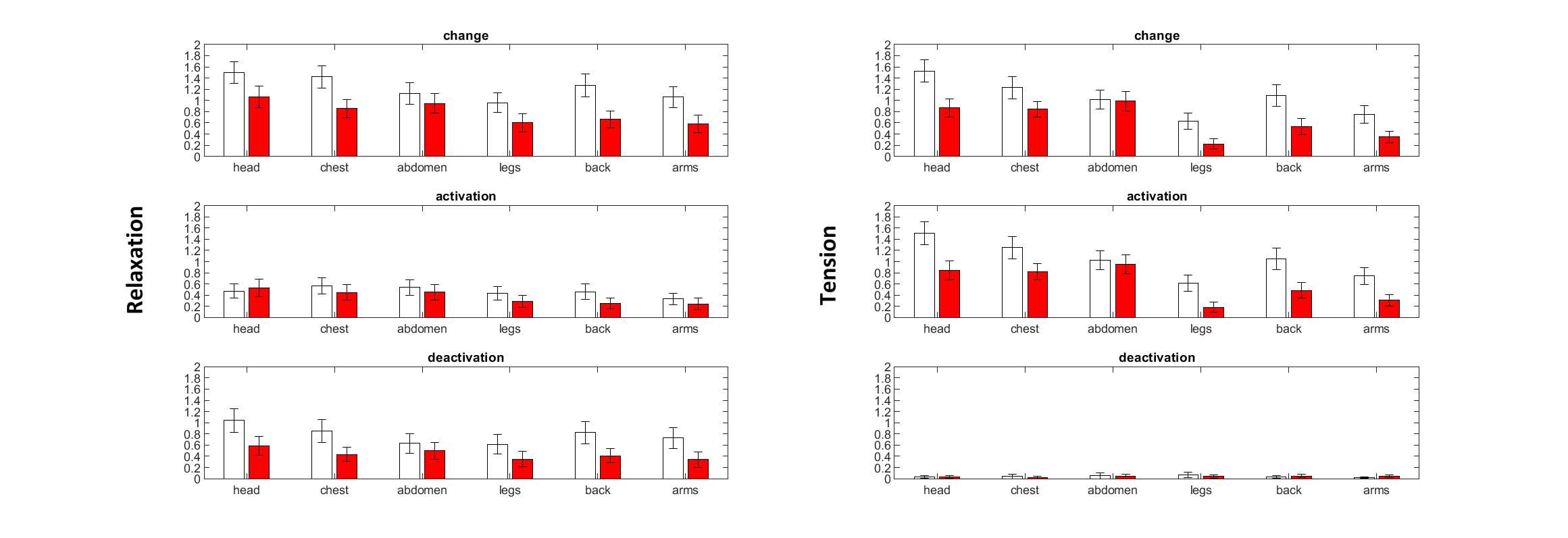


**Figure S4**. Means and standard deviations of the reported overall perceived bodily changes, activation, and deactivation in all pre-defined regions of interest for relaxation (left plot) and tension (right plot). Red bars indicate IBD group, white bars indicate HC group.

**Table S4.** Correlations between emotional awareness and general bodily changes

|  | **IBD** | | **HC** | | **IBD vs. HC** | |
| --- | --- | --- | --- | --- | --- | --- |
|  | *r_s_* | *p* _FDR_ | *r_s_* | *p* _FDR_ | *Z* | *p* |
| **Emotional awareness** |  |  |  |  |  |  |
| *Valence* |  |  |  |  |  |  |
| Positive emotions | .461 | .004 * | .154 | .317 | 1.53 | .064 (*) |
| Negative emotions | .393 | .011 * | .322 | .066 (*) | 0.36 | .359 |
| *Arousal* |  |  |  |  |  |  |
| Relaxation | .310 | .049 * | .424 | .008 * | -0.59 | .279 |
| Tension | .356 | .044 * | .282 | .064 (*) | 0.37 | .357 |

FDR, false discovery rate, (*) *p* < .10, ** p* < .05

**Table S5.** Correlations between reported symptom severity and emotion-related bodily sensations

|  | **IBD** | | **HC** | | **IBD vs. HC** | |
| --- | --- | --- | --- | --- | --- | --- |
|  | *r_s_* | *p* | *r_s_* | *p* | *Z* | *p* |
| **Anxiety (BSI-18)** |  |  |  |  |  |  |
| *Valence: Activation* |  |  |  |  |  |  |
| Positive emotions | .272 | .103 | .055 | .728 | 0.94 | .055 (*) |
| Negative emotions | **.563** | **.001 **** | .100 | .522 | **2.26** | **.012 *** |
| *Arousal: Activation* |  |  |  |  |  |  |
| Relaxation | -.039 | .818 | -.152 | .332 | 0.48 | .316 |
| Tension | **.465** | **.004 **** | .011 | .945 | 2.07 | .019 |
| *Valence: Deactivation* |  |  |  |  |  |  |
| Positive emotions | -.037 | .830 | .080 | .611 | -0.49 | .311 |
| Negative emotions | .208 | .216 | .080 | .609 | 0.55 | .291 |
| *Arousal: Deactivation* |  |  |  |  |  |  |
| Relaxation | **.384** | **.019 *** | .287 | .062 (*) | 0.46 | .323 |
| Tension | -.013 | .941 | -.064 | .682 | 0.22 | .415 |
| **Depression (BSI-18)** |  |  |  |  |  |  |
| *Valence: Activation* |  |  |  |  |  |  |
| Positive emotions | .194 | .257 | .173 | .268 | 0.09 | .464 |
| Negative emotions | **.562** | **.001 **** | .123 | .431 | **2.15** | **.016 *** |
| *Arousal: Activation* |  |  |  |  |  |  |
| Relaxation | .117 | .495 | -.052 | .743 | 0.71 | .238 |
| Tension | **.417** | **.011 *** | -.083 | .596 | **2.22** | **.013 *** |
| *Valence: Deactivation* |  |  |  |  |  |  |
| Positive emotions | .191 | .263 | .220 | .157 | -0.13 | .449 |
| Negative emotions | .235 | .168 | .208 | .180 | -0.29 | .388 |
| *Arousal: Deactivation* |  |  |  |  |  |  |
| Relaxation | **.437** | **.008 **** | **.311** | **.042 *** | 0.62 | .269 |
| Tension | -.036 | .836 | -.017 | .916 | -0.08 | .468 |

(*) *p* < .10, ** p* < .05, ** *p* < .01

**Table S6.** Results of the exploratory 2x2x2 mixed-effects ANOVA designs with factors “group” (IBD/HC), “sex” (female/male) and “valence” (positive/negative)/ “arousal” (relaxation/tension)

|  | *Dimension: Valence* | | | *Dimension: Arousal* | | |
| --- | --- | --- | --- | --- | --- | --- |
|  | *F* _1,81_ | *p*-value | | *F* _1,81_ | *p*-value | |
| Group | 4.23 | .043 | * | 7.08 | .009 | ** |
| Sex | 0.01 | .909 |  | 0.22 | .639 |  |
| Dimension | 0.15 | .700 |  | 0.53 | .469 |  |
| Group*Sex | 2.82 | .097 |  | 0.62 | .432 |  |
| Group*Dimension | 0.01 | .909 |  | 0.23 | .633 |  |
| Sex*Dimension | 0.74 | .392 |  | 0.02 | .885 |  |
| Group*Sex*Dimension | 0.66 | .419 |  | 1.57 | .214 |  |

** p* < .05, ** *p* < .01

**Table S7**. Results of the exploratory 2x2x2x2 mixed-effects ANOVA designs with the factors “group”, “sex”, “valence”/ “arousal” and “type of change”

|  | *Dimension: Valence* | | | *Dimension: Arousal* | | |
| --- | --- | --- | --- | --- | --- | --- |
|  | *F* | *p*-value | | *F* | *p*-value | |
| Group | 6.44 | .013 | * | 13.07 | < .001 | *** |
| Sex | 0.02 | .889 |  | 0.74 | .391 |  |
| Dimension | 2.87 | .092 |  | 2.29 | .132 |  |
| Type of change | 87.40 | < .001 | *** | 14.59 | < .001 | *** |
| Group*Sex | 4.92 | .029 | * | 6.45 | .013 | * |
| Group*Dimension | 0.12 | .730 |  | 0.10 | .756 |  |
| Sex*Dimension | 4.21 | .041 | * | 0.11 | .738 |  |
| Group*Type of change | 0.97 | .327 |  | 7.33 | .007 | ** |
| Sex*Type of change | 0.47 | .493 |  | 1.51 | .221 |  |
| Dimension*Type of change | 13.08 | < .001 | ******* | 45.63 | < .001 | *** |
| Group*Sex*Dimension | 0.73 | .394 |  | 0.28 | .600 |  |
| Group*Sex*Type of change | 5.51 | .020 | ***** | 1.55 | .215 |  |
| Group*Dimension*Type of change | 6.02 | .015 | ***** | 11.49 | < .001 | *** |
| Sex*Dimension*Type of change | 5.26 | .023 | ***** | 3.20 | .075 |  |
| Group*Sex*Dimension*Type of change | 0.05 | .826 |  | 12.41 | < .001 | *** |

** p* < .05, ** *p* < .01, *** *p* < .001


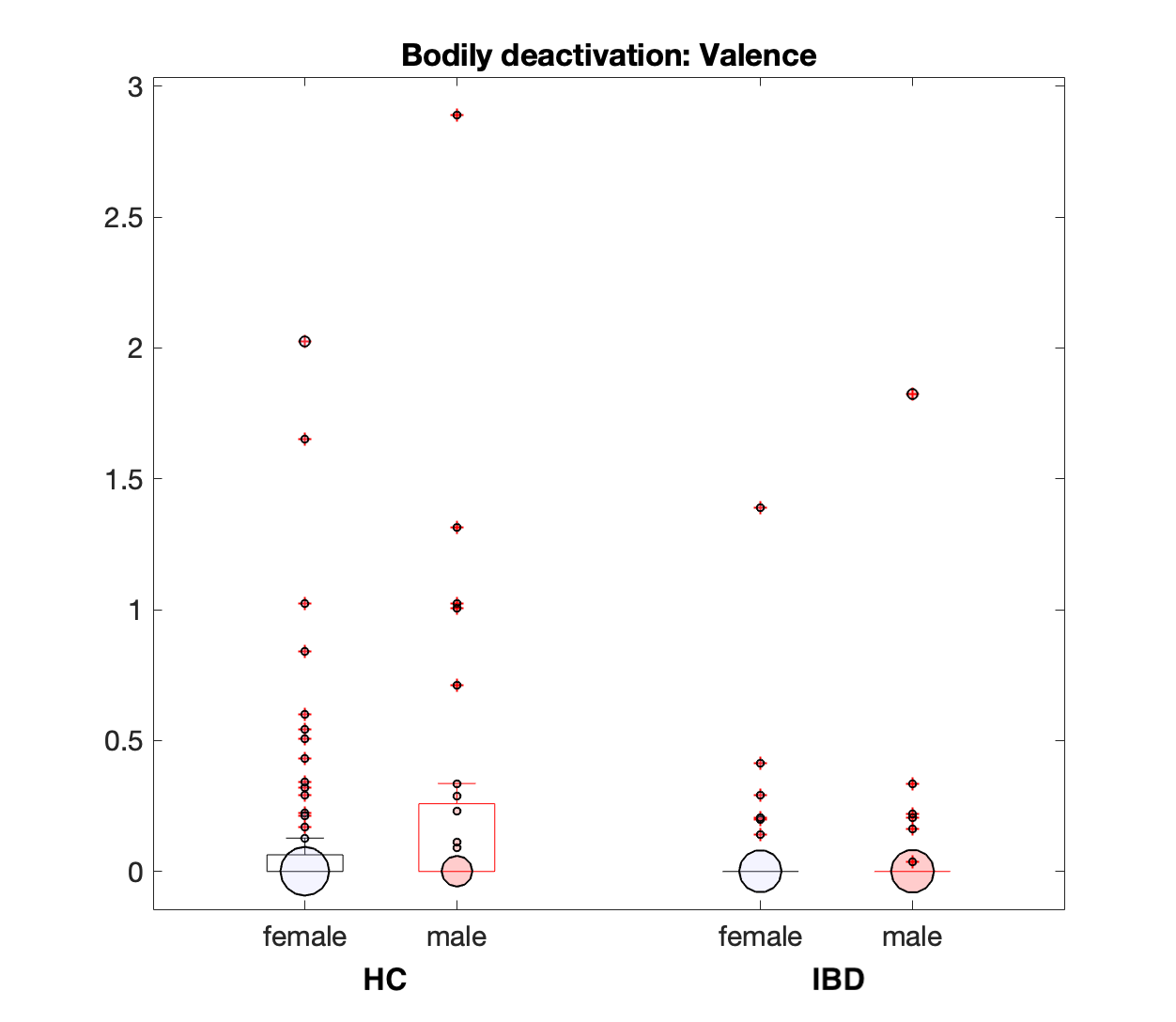
**Figure S5.** Group differences in the reported bodily deactivation scores associated with the experience of valence dependently on participants’ sex.


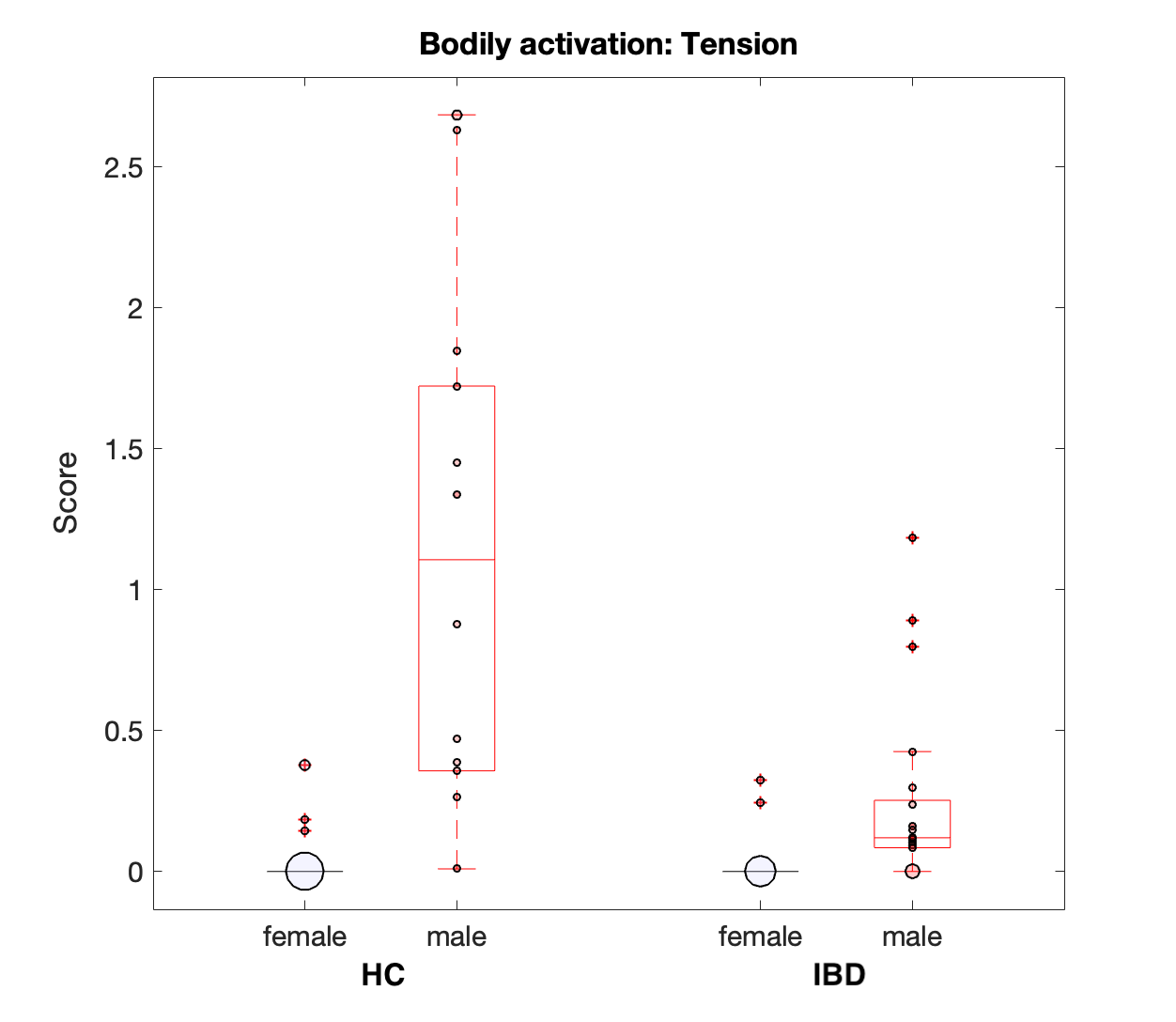


**Figure S6.** Group differences in the reported bodily activation scores associated with the experience of tension dependently on participants’ sex.


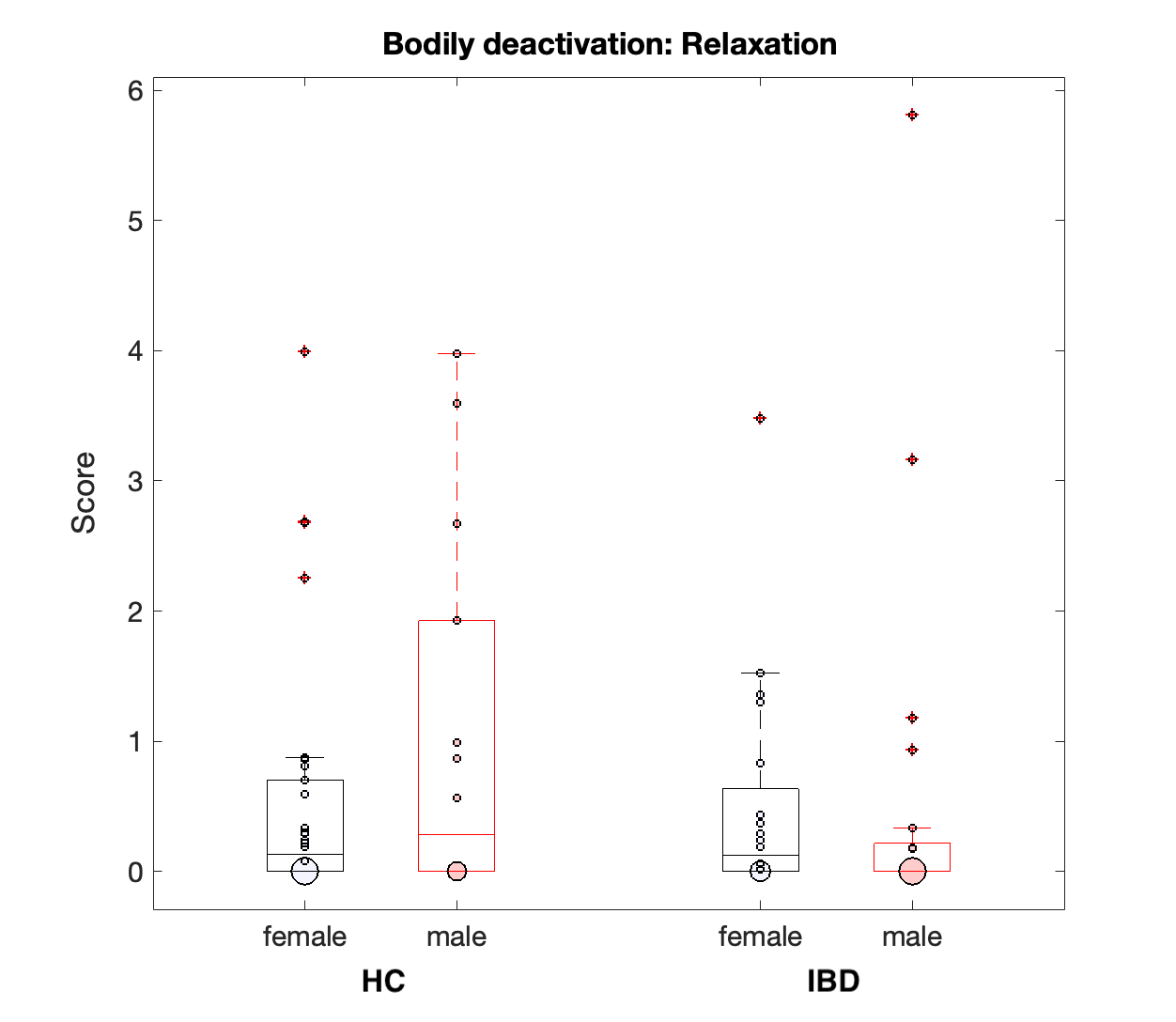


**Figure S7.** Group differences in the reported bodily deactivation scores associated with the experience of relaxation dependently on participants’ sex.
